# Gallstone ileus causing perforation of multiple segments of small bowel

**DOI:** 10.1093/jscr/rjae647

**Published:** 2024-10-19

**Authors:** Andrew D Eiref, Karri Hester, Tyler Glaspy, Anna Sarkisova, Sohini Anand, Patrick W Zimmerman, Michael Nicoara, Krishan Patel, Heath Walden, Simon D Eiref

**Affiliations:** Department of Surgery, Department of Pathology, Danbury Hospital, 24 Hospital Ave, Danbury, CT 06810, United States; Department of Surgery, Department of Pathology, Danbury Hospital, 24 Hospital Ave, Danbury, CT 06810, United States; Department of Surgery, Department of Pathology, Danbury Hospital, 24 Hospital Ave, Danbury, CT 06810, United States; Department of Surgery, Department of Pathology, Danbury Hospital, 24 Hospital Ave, Danbury, CT 06810, United States; Department of Surgery, Department of Pathology, Danbury Hospital, 24 Hospital Ave, Danbury, CT 06810, United States; Department of Surgery, Department of Pathology, Danbury Hospital, 24 Hospital Ave, Danbury, CT 06810, United States; Department of Surgery, Department of Pathology, Danbury Hospital, 24 Hospital Ave, Danbury, CT 06810, United States; Department of Surgery, Department of Pathology, Danbury Hospital, 24 Hospital Ave, Danbury, CT 06810, United States; Department of Surgery, Department of Pathology, Danbury Hospital, 24 Hospital Ave, Danbury, CT 06810, United States; Department of Surgery, Department of Pathology, Danbury Hospital, 24 Hospital Ave, Danbury, CT 06810, United States

**Keywords:** gallstone ileus, small bowel obstruction, intestinal perforation, cholecystoduodenal fistula

## Abstract

Gallstone ileus results in a mechanical small bowel obstruction when an itinerant gallstone tumbles downstream and obstructs the bowel lumen. Associated proximal intestinal injury with perforation is rare, and concomitant perforation of multiple segments of bowel in the setting of gallstone ileus has never been reported in the literature. We are reporting the case of a 67-year-old female patient who had gallstone ileus causing perforation of multiple segments of small bowel. At operation, she was found to have a 3.2-cm gallstone lodged at the terminal ileum, perforation of both the mid ileum and mid jejunum, and gross enteric spillage. She underwent removal of the gallstone and small bowel resection × 2. She was initially left in discontinuity with an open abdomen. She returned to the operating room 2 days later for bowel anastomosis and abdominal closure. She recovered well after surgery and was discharged home.

## Introduction

Gallstone ileus is a rare complication of cholelithiasis that presents most commonly in female and elderly patients above the age of 65 years, and accounts for 1%–4% of cases of mechanical small bowel obstruction (SBO) [1–3]. Gallstone ileus results when cholelithiasis causes recurrent inflammation and erosion of the gallbladder wall, with fistulization to adjacent bowel [3, 4]. Passage of a gallstone into the bowel lumen is usually via connection between the gallbladder and duodenum, with cholecystoduodenal accounting for 85% of biliary-enteric fistulas [3]. Subsequent journey of the gallstone downstream may be characterized by a tumbling phenomenon with alternating episodes of partial obstruction and distal migration [2, 5]. This journey usually comes to an end in the terminal ileum where the lumen narrows and 60% of the stones become firmly impacted, causing mechanical SBO [3]. Gallstone ileus with associated intestinal perforation proximal to the area of obstruction is rare with only 10 reported cases in the literature [6, 7]. These cases involved perforation of a single segment of bowel [4–9], whereas concomitant perforation of multiple segments of bowel has never been reported. Here, we describe the presentation and operative management of the first documented case of gallstone ileus causing perforation of multiple segments of small bowel.

## Case report

**Figure 1:**
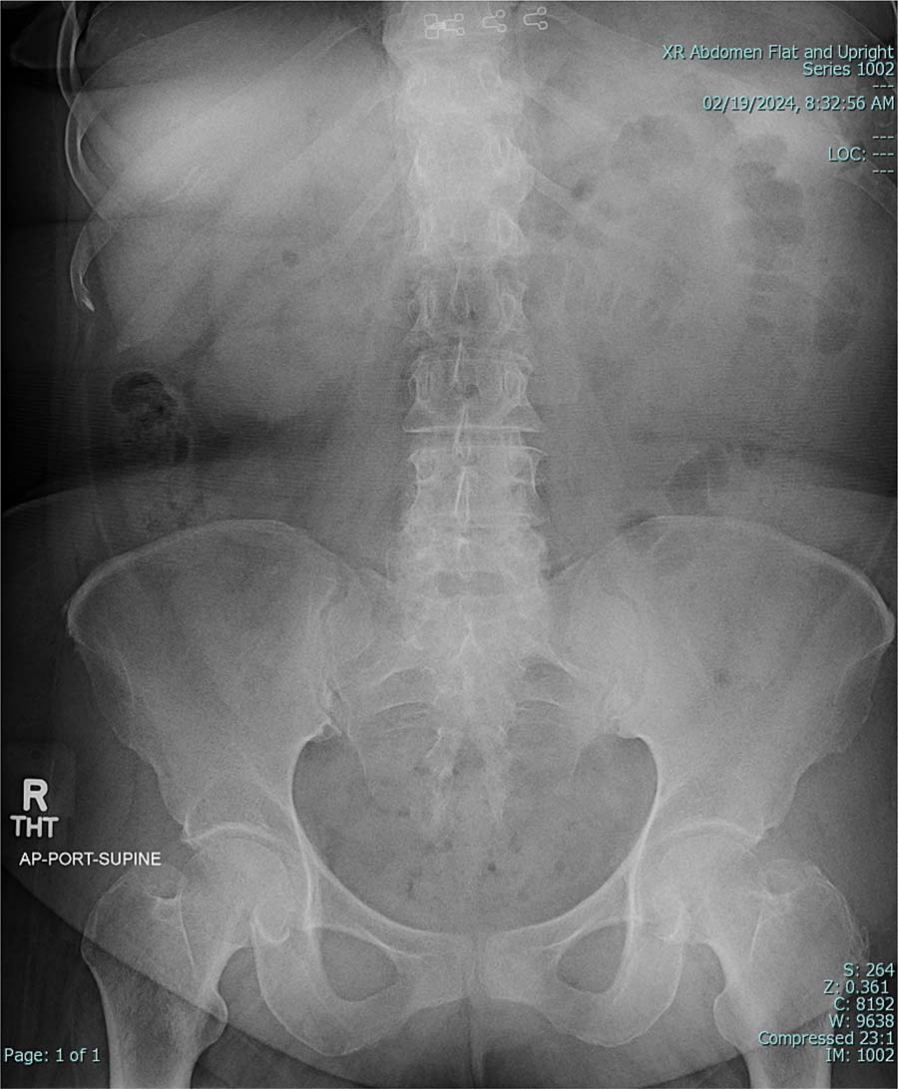
Preoperative X-ray of abdomen, demonstrating small bowel obstruction.

**Figure 2:**
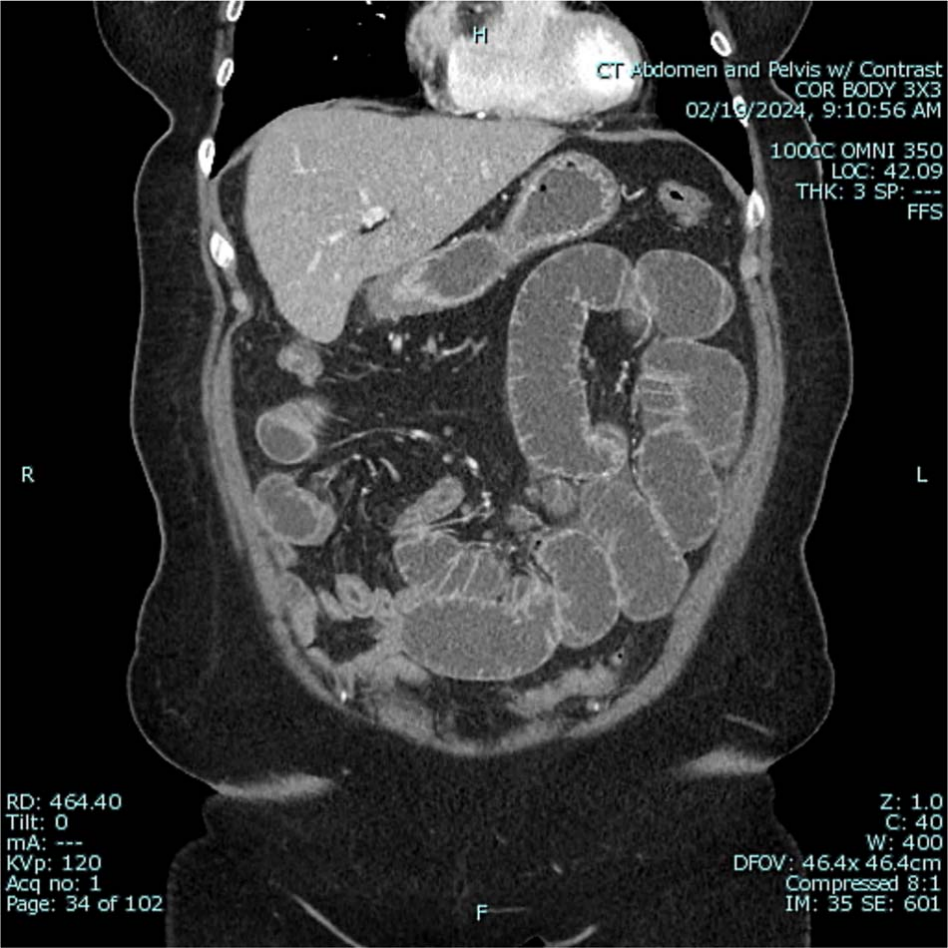
Preoperative CT of abdomen in coronal view, demonstrating small bowel obstruction.

A 67-year-old female patient with a past medical history significant only for C-section, presented with 1 week of intermittent abdominal pain, distension, and nausea with vomiting. Vital signs were in the normal range. Physical exam was notable for a soft and nondistended abdomen with right lower quadrant tenderness. Laboratory studies were unremarkable. Radiological imaging (Figs 1 and 2) demonstrated a developing SBO.

**Figure 3:**
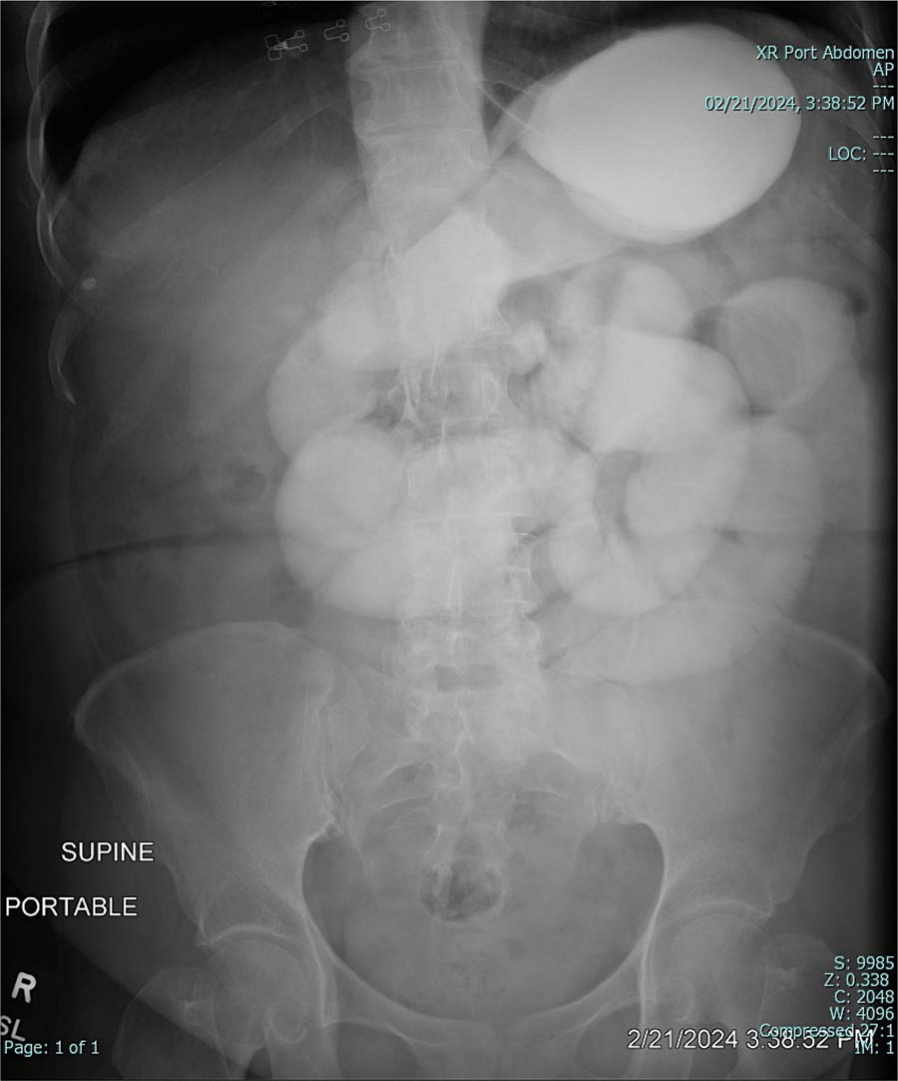
Gastrografin challenge reveals passage of contrast through the small intestine and into the colon on sequential imaging [300 × 361 mm (72 × 72 DPI)].

**Figure 4:**
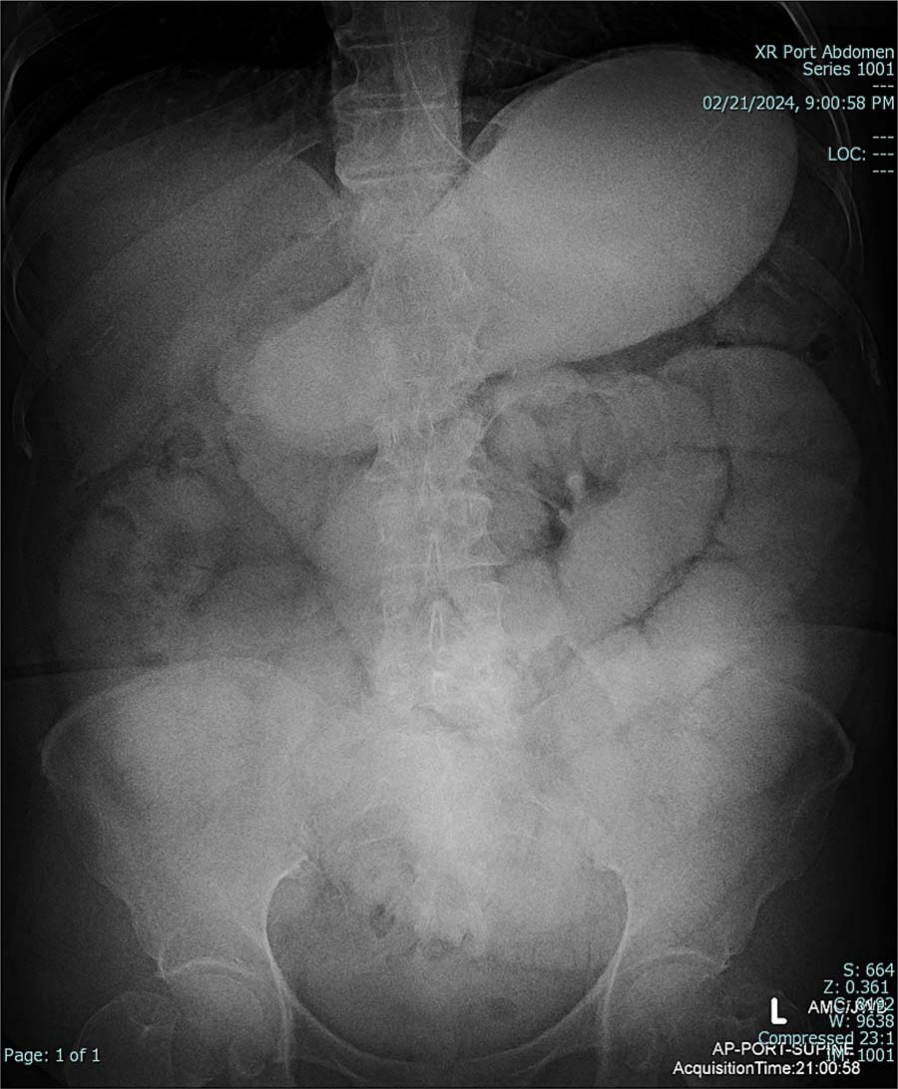
Gastrografin challenge reveals passage of contrast through the small intestine and into the colon on sequential imaging [297 × 360 mm (72 × 72 DPI)].

The patient was admitted to the hospital for conservative management of suspected adhesive SBO. She was treated with intravenous fluid hydration and nasogastric tube decompression. On hospital days 2–4, she began to feel better and pass flatus. Gastrografin challenge revealed passage of contrast through the small intestine and into the colon (Figs 3 and 4). On hospital day 5, however, she had cessation of bowel function with worsening abdominal pain, tachycardia, and leukocytosis (WBC 16 K/μl).

**Figure 5:**
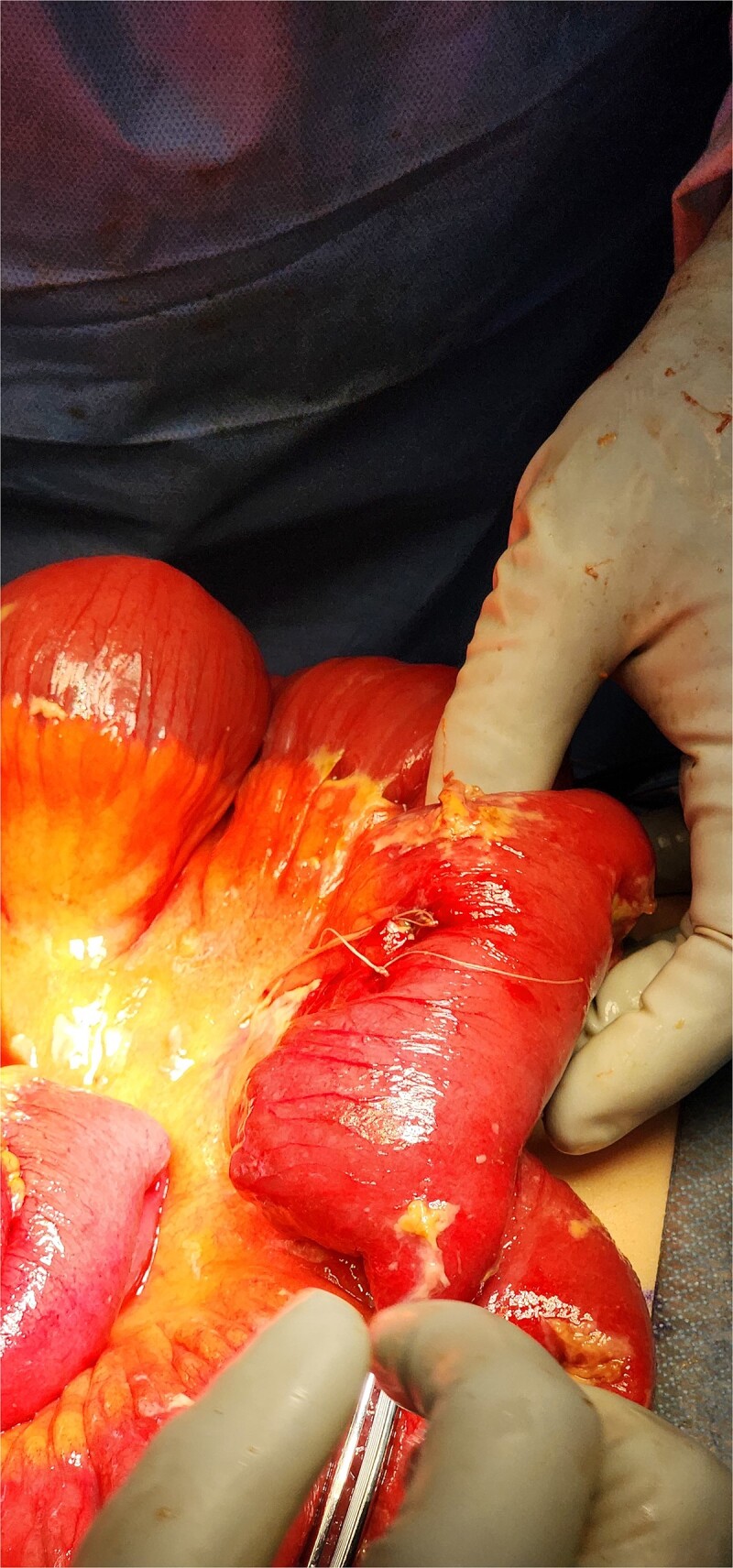
Intraoperative photo of segment of ileum, demonstrating area of perforation.

**Figure 6:**
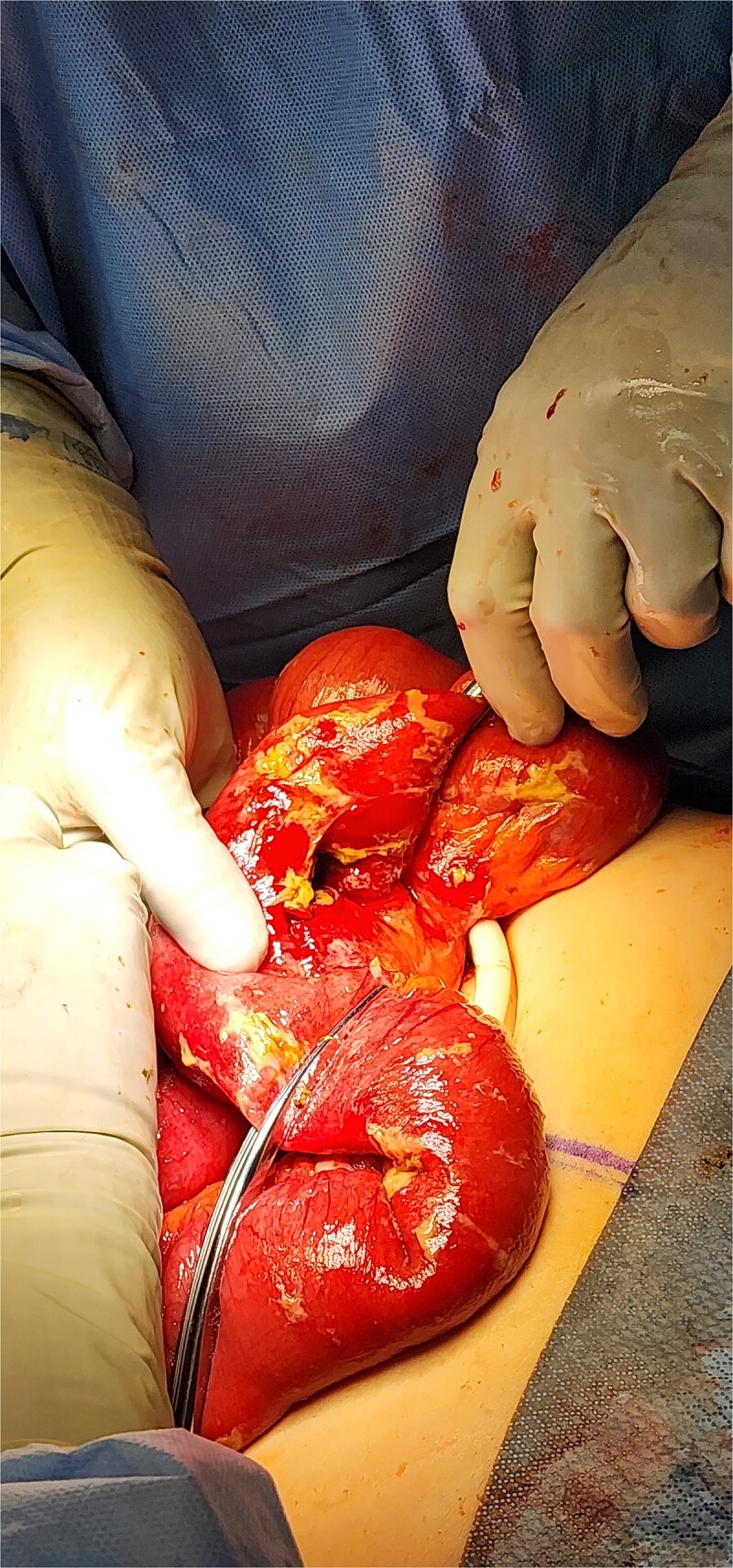
Intraoperative photo of segment of jejunum, demonstrating area of perforation.

**Figure 7:**
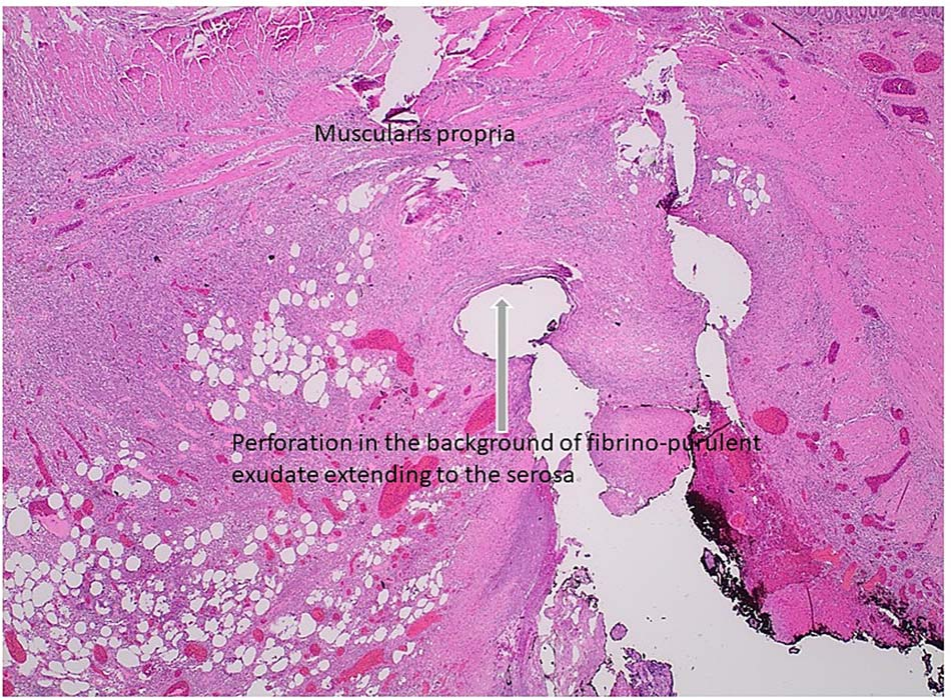
Histologic section of intestinal perforation site that is inked black with H&E stain, suggesting a pressure-related injury.

**Figure 8:**
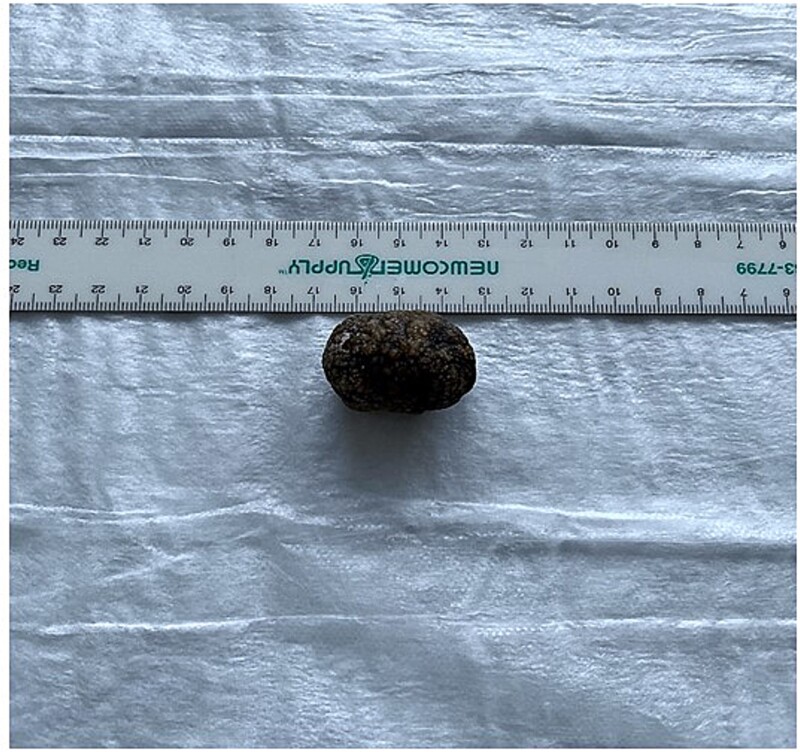
Gross examination of the gallstone.

The patient was taken to the operating room. Diagnostic laparoscopy revealed free succus with surrounding inflammation and fibrinous exudate. Conversion to exploratory laparotomy revealed a hard, ovoid mass lodged in the terminal ileum that was causing an SBO, and perforations in multiple segments of small bowel proximal to the obstruction. There were two perforations of the mid-ileum (Fig. 5) and three perforations of the mid-jejunum (Fig. 6). In each segment, the perforations were clustered closely together (Fig. 7). The obstructing mass was milked back into the mid-ileum in preparation for removal. The perforated mid-ileum containing the mass and perforated mid-jejunum both underwent bowel resection. The specimen was opened on the back table revealing a 3.2-cm diameter intraluminal gallstone (Supplementary Video 1, Fig. 8).

**Figure 9:**
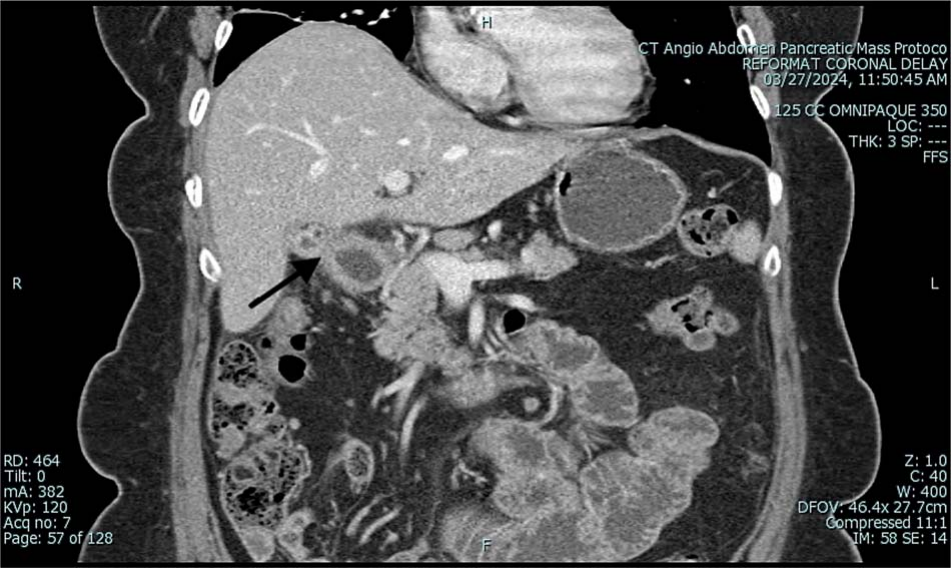
Postoperative CT of abdomen in coronal view, demonstrating suspected cholecystoduodenal fistula.

Over the course of the operation, the patient became septic and was started on vasopressor support. Her surgery was expedited by leaving the bowel in discontinuity and placing a wound vac for temporary abdominal closure. After stabilization, she returned to the operating room on hospital day 7 for bowel anastomosis and definitive abdominal closure. She recovered well postoperatively and was discharged home on hospital day 18. Follow-up computed tomography (CT) revealed a cholecystoduodenal fistula (Fig. 9). Elective surgery for cholecystectomy and fistula repair is being considered.

## Discussion

Gallstone ileus results in mechanical SBO and rarely intestinal perforation. This is the first reported case of gallstone ileus causing perforation of multiple segments of small bowel. Our case highlights the fact that gallstone ileus is a morbid disease that can be difficult to diagnose, and whose surgical management is evolving.

Gallstone ileus has a high mortality of 7%–30% [3]. Gallstone ileus generally affects patients who are elderly, frail, and have multiple medical comorbidities [3]. These patients have less physiological reserve and greater medical risk, with 86% having American Society of Anesthesiologists classification 3 or 4 at the time of surgery [6].

Symptoms of gallstone ileus can be vague and insidious leading to delay in seeking medical care on average between 4 and 8 days [3, 7]. Signs can be nonspecific and diagnosis challenging, with delay between hospital admission and surgical intervention ranging from 2 to 37 days [3]. In the interim, progression of disease can lead to a higher severity of illness and worse outcome.

Our patient was typical in that she was an elderly female presenting for hospital admission late after the onset of symptoms. Classic complaints of intermittent abdominal pain, nausea, and vomiting likely resulted from alternating episodes of partial obstruction and distal migration as the gallstone tumbled downstream.

As in our patient, the diagnosis is often confirmed only at the time of laparotomy [10]. Preoperative imaging with X-ray has a 40%–70% and CT has a 93% sensitivity in diagnosing gallstone ileus [1, 3]. In particular, visualizing the Rigler triad of pneumobilia, SBO, and ectopic gallstone is pathognomonic [4]. We suspect that our patient’s gallstone was either pure cholesterol or composite in composition making it harder to detect radiographically [3].

Injury to the mid-ileum and mid-jejunum was likely initiated by gallstone-induced pressure necrosis of these areas during periods of partial obstruction as the gallstone tumbled downstream. Subsequent intestinal blowout and perforation were likely secondary to the added effect of high intraluminal pressure, developing once the gallstone had become firmly impacted at the terminal ileum [5–7].

Management of gallstone ileus has evolved from a one-stage to a two-stage surgical approach. The one-stage procedure combines enterolithotomy, cholecystectomy, and fistula repair. The two-stage procedure consists of initial enterolithotomy alone and subsequent surgery for cholecystectomy and fistula repair as needed [2].

The two-stage procedure has lower morbidity and mortality [7]. In addition, recent studies have shown the possibility of spontaneous fistula closure after enterolithotomy alone in up to 60% of cases [1]. We are evaluating whether further biliary surgery will be needed in our patient based upon the results of serial CT.

## Supplementary Material

Supplementary_Video_1_rjae647
